# Sinisan Protects Primary Hippocampal Neurons Against Corticosterone by Inhibiting Autophagy via the PI3K/Akt/mTOR Pathway

**DOI:** 10.3389/fpsyt.2021.627056

**Published:** 2021-05-28

**Authors:** Mingjia Zhang, Yi Zhang, Haitao Sun, Hui Ni, Jialing Sun, Xuemei Yang, Weicong Chen, Wenting Zhao, Xiaodan Zhong, Chunyu He, Haiqing Ao, Songqi He

**Affiliations:** ^1^School of Traditional Chinese Medicine, Southern Medical University, Guangzhou, China; ^2^Department of Psychology, School of Economics and Management, Guangzhou University of Chinese Medicine, Guangzhou, China; ^3^Guangdong Food and Drug Vocational College, Guangzhou, China

**Keywords:** sinisan, primary hippocampal neurons, corticosterone, autophagy, depression

## Abstract

**Objective:** Corticosterone causes significant neurotoxicity in primary hippocampal neurons which is associated with depression. Dysfunctional autophagy is implicated in cognitive impairment and depressive-like behavior. The traditional Chinese medicine Sinisan (SNS) is highly effective in clinical treatment of depression. However, the molecular mechanisms underlying therapeutic effects of SNS are unknown.

**Purpose:** The aim of this study was to elucidate the protective effect of SNS and the underlying mechanisms against corticosterone-induced neuronal damage.

**Study Design:** The effects of serum derived from rats containing SNS (or untreated controls) on the expression of autophagy-related molecules in primary rat hippocampal neurons exposed to different concentrations of corticosterone for different intervals were explored.

**Methods:** CCK-8 assay, LDH assay were used to analyze cell viability and LDH activity. Western blot, qRT-PCR, and immunofluorescence assays were used to determine protein and mRNA expression levels of molecules such as LC3, p62, Beclin1, ULK1, PI3K, p-PI3K, Akt p-Akt, mTOR, p-mTOR, p70S6, p-p70S6, 4ebp1 and p-4ebp1.

**Results:** Corticosterone induced a dose- and time-dependent reduction in cellular viability. Moreover, corticosterone (100–400 μM) treatment for 24 h increased LC3-II/LC3-I protein ratio, increased Beclin1 and ULK1 protein expression levels, and decreased p62, PI3K, p-PI3K, p-Akt, p-mTOR, p-p70S6, and p-4ebp1 protein expression levels. Notably, SNS-containing serum reversed corticosterone-induced reduction of neuronal viability, and increased p62, PI3K, p-Akt, p-mTOR, p-p70S6, and p-4ebp1 protein and mRNA expression levels. In addition, SNS-containing serum decreased LC3-II/LC3-I protein ratio, and downregulated Beclin1, and ULK1 protein and mRNA expression in primary hippocampal neurons.

**Conclusion:** SNS protects primary hippocampal neurons against corticosterone-induced neurotoxicity by preventing excessive autophagy through activation of PI3K/AKT/mTOR pathway.

## Introduction

Depression is a condition characterized by low mood, loss of interest, low self- esteem, and suicidal impulse. Depression is one of the most common mental conditions worldwide (1, 2). Incidence of depression worldwide has increased by more than 18% between 2005 and 2015, and currently over 300 million people are living with depression. However, the cause of increase in prevalence of depression are unknown. Excessive stress plays an important role in development of depression (3, 4). Hyperactivity of the hypothalamic Pituitary Adrenal (HPA) axis is implicated in depression. Hippocampus is the major target of corticosterone which is the final product of HPA axis, and is correlated with pathogenesis and progression of depression (5). Therefore, we hypothesized that drugs used for inhibition of corticosterone-induced neurotoxicity in the hippocampus can be applied for clinical treatment of depression. Use of antidepressants is associated with various side effects such as cardiac toxicity, sexual dysfunction, obesity, and insomnia. Moreover, the rate of successful treatment of depression is approximately 70% (6, 7). Therefore, studies should explore better tolerated and more effective drugs for treatment of depression.

Traditional Chinese medicine (TCM) has been used successfully for treatment of depression. Sinisan decoction (SNS) reported by Zhongjing Zhang in “Treatise on Febrile Diseases” in 200–201 AD contains Bupleuri Radix (BR), Paeoniae Radix Alba (PRA), Aurantii Fructus Immaturus (AFI), and Glycyrrhizae Radix et Rhizoma Praeparata Cum Melle (GRM) (8). SNS is regarded as an effective and essential anti-depression therapy in TCM (9, 10). SNS treatment improves weight loss, activity in open field test (OFT), and sucrose preference test (SPT) performance in mouse models with depressive-like behavior (11). However, the mechanisms underlying the anti-depressive effects of SNS have not been explored.

Macro-autophagy (hereafter referred to as autophagy) is implicated in protein degradation in cells. In autophagy process, target proteins are packaged in a double-membrane vesicle (autophagosome), which fuses with lysosomes for degradation of its contents. Autophagy is a cell survival mechanism involved in removal of damaged cytosolic organelles and misfolded proteins to maintain homeostasis. Dysfunctional autophagy results in a variety of pathological processes. Prenatal stress induces cognitive impairment and depressive-like behavior through autophagy (12). Previous study reported that inhibition of autophagy prevents depressive-like behavior induced by ecstasy in rats (13).

PI3K/Akt/mTOR pathway is implicated in modulation of hippocampal activities that result in long-term depression (14). Eight-week chronic unpredictable mild stress (CUMS) exposure downregulates mTOR and the upstream and downstream signaling proteins (15). Therefore, PI3K/Akt/mTOR pathway is associated with depression (16).

In this study, primary hippocampal neurons were used as an *in vitro* system to explore the effects of SNS treatment on corticosterone-induced neuronal damage and elucidate the underlying mechanisms.

## Materials and Methods

### Animals

Adult male (8 weeks old) and newborn Sprague-Dawley (SD) rats were obtained from Guangzhou University of Chinese Medicine. Adult animals were housed under normal environmental conditions (12 h light/dark cycle; lights on at 8:00 a.m., 20 ± 2°C). Rats were housed in plastic cages at a density of 5 per cage with free access to food and tap water. Animals were allowed to acclimatize for a week prior to experimental sessions. All animal experiments were approved by the Animal Care and Committee of Guangzhou University of Chinese Medicine. Experimental protocols were designed to minimize animal suffering.

### Primary Hippocampal Neuron Culture and Treatment

Primary hippocampal neurons were obtained from newborn SD rats (~1 day old). Animals were euthanized and immediately hippocampus from each animal was separated and dissected into 0.5-mm pieces in DMEM-F12 (Gibco, America). After harvesting of hippocampus, the meninges were gently removed under sterile conditions. The samples were treated with 0.125% trypsin (Gibco, USA) at 37°C for 30 min with continuous shaking. Samples were further suspended in DMEM-F12 containing 10% FBS (Gibco, America). Disassociated neurons were centrifuged at 152 g for 5 min and loaded at a density of 1.5 × 10^5^-1 × 10^6^ cells/well on poly-d-lysine-coated plates (sigma, America) for cell viability, immunofluorescence, western blot, and qRT-PCR analysis. Cells were initially maintained in DMEM-F12 containing 10% FBS. At 4 h post-plating, the media was replaced with neurobasal media (Gibco, USA) containing 2% B27 (Gibco, USA) and 1% L-glutamine (Gibco, USA). Cells were cultured at 37°C in a humidified 5% CO2 incubator, and half of the medium was changed every 2 days.

Primary hippocampal neurons were cultured for 7 days, then different concentrations and time of corticosterone were incubated to determine the appropriate damaging concentration of corticosterone. Neurons were then grouped including untreated (control group), corticosterone group, corticosterone + normal serum group, and corticosterone + SNS-containing serum group to explore the protective effect of SNS on corticosterone-induced neurons. Cells in the corticosterone + normal serum group and corticosterone + SNS-containing serum group were incubated in serum with corticosterone for 24 h. Normal serum and SNS-containing-serum were administered for 0.5 h prior to treatment with corticosterone.

### Preparation of Chinese Herbal Solutions

SNS concentrate particles were provided by the Department of Guangdong Yi Fang Pharmaceutical Company (Guangdong, China). Concentrate particles comprised 12 g Bupleuri Radix (BR), 12 g Paeoniae Radix Alba (PRA), 12 g Aurantii Fructus Immaturus (AFI), and 12 g Glycyrrhizae Radix et Rhizoma Praeparata Cum Melle (GRM). Concentrate particles were dissolved, sealed, and stored at 4°C. The concentration of crude concentrations of SNS solutions was 0.49 g/mL.

### Drug-Containing Serum Preparation

Twenty SD rats were randomly divided into normal serum and SNS groups (*n* = 10 per group). SNS (2 mL, once a day) was administered to rats in the SNS group by gavage methods for seven days. Equal volumes of physiological saline solution were administered to rats in the normal serum group for seven successive days. Rats were anesthetized using sodium pentobarbital (40 mg/kg) 2 h after the last drug administration. Blood was collected through the abdominal aorta, centrifuged at 1,452 g for 10 min, and allowed to stand at 4°C for 4 h. Supernatant serums of the same group were pooled, filtered using a 0.22 μmol/L filter, inactivated at 56°C for 30 min, split into several samples, and stored at −20 °C.

### Small Interfering RNA (si-RNA)

Target sequences were 5′-GCAAGACACC ATGAACCAT-3′ for mTOR α1 sense strand, 5′-CCAAAGCACTACACTACAA-3′ for mTOR α2, and 5′-GCTAGAAGCCTTTGTCTAT-3′ for mTOR α3. The target sequence for the mTOR α3 sense strand was selected. A nonspecific siRNA sequence was used as negative control. Transfection reagents (Ribobio, China) were prepared using riboFECTTM CP Transfection Kit (Ribobio, China) following the manufacturer's instructions. Neurons were transfected with 50 nM siRNA-mTOR (Ribobio, China). After transfection for 24 h, mTOR protein expression levels were determined using western blot.

### CCK-8 Assay

Cell Counting Kit-8 (CCK-8) assay was used to determine the viability of primary hippocampal neurons following the protocol of the kit. Neurons were washed 3 times with PBS (Gibco, America). After washing, 100 μL neurobasal medium and 10 μL CCK-8 solution (DOJINDO, Japan) were added to each well. The plates were then incubated for 1.5 h at 37°C under dark conditions. A microplate reader (Bio-Rad, America) was used to determine the optical density (OD) for each well at 570 nm.

### LDH Assay

Neuronal injury was evaluated by determination of LDH activity 24 h after administration of corticosterone and drug-containing serum, using colorimetric assay (Solarbio, china). Absorbance was determined at a wavelength of 450 nm to measure LDH activity and determine the number of damaged cells following the manufacturer's instructions. Data were normalized based on LDH activity of the control culture media (100%).

### Immunofluorescence

Cells were loaded in 12 well-plate, washed 3 times and neurons fixed with 4% paraformaldehyde (Meilun, China) for 15 min at room temperature. Samples were then washed 3 times, blocked and permeabilized with 5% normal goat serum in PBS (PBS containing 0.2% BSA (Roche, America) and 0.2% Triton X-100 (Solarbio, China) for 30 min (PBS/BSA/Triton). Primary antibodies against LC3 (CST, USA) were diluted to 1:200 in PBS/BSA/Triton and added to samples and were incubated overnight at 4°C. Samples were then washed 3 times for 10 min and incubated with Alexa-Fluor 488-labeled secondary antibodies (CST, USA) diluted to 1:500 in PBS/BSA/Triton for 1 h in the dark. After washing the samples 3 times for 10 min, nuclei were counterstained with DAPI stain diluted to 1:1,000 PBS (Beyotime, China) for 5 min. Coverslips were washed 3 times for 10 min and mounted on glass slides with Polyvinylpyrrolidone (Beyotime, China). Immunofluorescence was determined using laser scanning confocal microscope (ZEISS, Germany).

### Western Blot

After removal of culture medium, neurons were washed three times with PBS, and immediately lysed using 1 ml of RIPA (Sigma, America) containing protease and phosphatase inhibitor cocktails (Roche, America) to obtain total proteins. BCA kit (Beyotime, China) was used to quantify proteins in the samples. For immunoblotting, equal amounts of proteins were separated by 8–16% SurePageTM Gels (GenScript, USA), transferred to polyvinylidene difluoride membrane (Roche, America) and then blocked for 1 h using 5% BSA diluted in TBS-T. Membranes containing proteins were incubated with LC3, Beclin1, p62, ULK1, PI3K, p-PI3K, Akt, p-Akt, mTOR, p-mTOR, p70S6, p-p70S6, 4ebp1, p-4ebp1, β-actin (1:2,000; all from CST, America) primary antibodies overnight. Membranes were then incubated with the corresponding secondary HRP-coupled anti-rabbit antibody (1:5,000; CST, America) for 2 h. ECL detection system (Tanon, China) was used to visualize bands. For quantitative analysis, the bands were analyzed using ImageJ software.

### qRT-PCR

Trizol (Invitrogen, America) was used to extract total cellular RNA following the manufacturer's instructions. Quality and quantity of RNA were determined using Nanodrop One (ThermoFisher, America) at 260 nm and 260/280 nm, respectively. One μg of total RNA was reverse transcribed to obtain cDNA using RevertAid First Strand cDNA Synthesis Kit (Invitrogen, USA) in the T100TM Thermal Cycler (Bio-rad, America). Quantitative gene expression was performed following the FastStart Universal SYBR@ Green Master (Invitrogen, America) protocol using a CFX96TM Real-Time PCR System (Bio-rad, America). Primer sequences used (Takara, Japan) for qRT-PCR are presented in Table 1. qRT-PCR data was analyzed using the 2^−ΔΔCt^ method with threshold cycle values.

**Table 1:** Sequences of qRT-PCR primers used.

**Gene**	**Primer (5**^****′****^**-3**^****′****^**)**	
β-actin	Forward:GGAGATTACTGCCCTGGCTCCTA	Reverse:GACTCATCGTACTCCTGCTTGCTG
mTOR	Forward:GCTTATCAAGCAAGCGACATCTCA	Reverse:TCCACTGGAAGCACAGACCAAG
LC3	Forward:AGCTCTGAAGGCAACAGCAACA	Reverse:GCTCCATGCAGGTAGCAGGAA
p62	Forward:AAGCTGCCCTGTACCCACATC	Reverse:ACCCATGGACAGCATCTGAGAG
P70S6	Forward:AGGATGCAGGCTCTGAGGA	Reverse:ACCAAGTACCCGAAGTAGCTCAA
Beclin1	Forward:GAAACTGGACACGAGCTTCAAGA	Reverse:ACCATCCTGGCGAGTTTCAATA
4ebp1	Forward:TCACTAGCCCTACCAGCGATGAG	Reverse:CCAGAAGCATCACTGCGTCCTAT
ULK1	Forward:CCACTGCGTGGCTCACCTAA	Reverse:TAGCCAACAGGGTCAGCAAACTC
AKT	Forwar:ATGGACTTCCGGTCAGGTTC	Reverse:GCCCTTGCCCAGTAGCTTCA

### Statistical Analysis

Experimental data were presented as mean ± S.E.M. of at least three independent experiments. Statistical analysis was performed using SPSS 23.0 software (SPSS, Chicago, IL, USA). Multiple group comparisons were performed using one-way analysis of variance (ANOVA) followed by Dunnett's test for inter-group differences. *P* < 0.05 was considered statistically significant.

## Results

### SNS Protects Primary Hippocampal Neurons Against Corticosterone Induced Neurotoxicity

**Figure 1:**
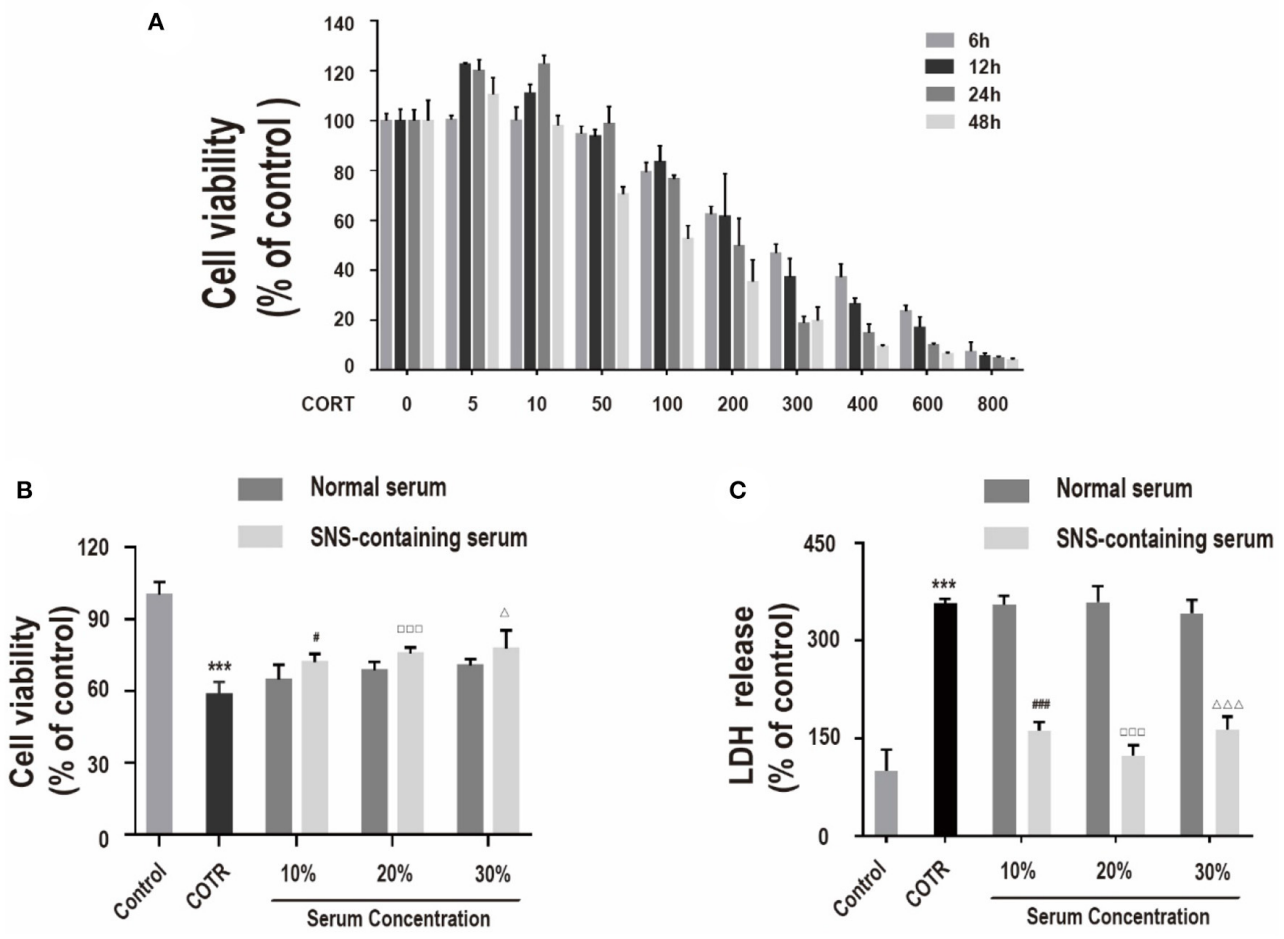
**(A)** Viability of cells incubated with different concentrations of corticosterone (0, 5, 10, 50, 100, 200, 300, 400, 600, 800 μM) for different intervals (6, 12, 24, 48 h), determined by CCK-8 assay. (*n* = 4). **(B)** Viability of cells co-incubated with 200 μM corticosterone and 10%, 20%, or 30% SNS-containing serum for 24 h. Each column represents mean ± SD (*n* = 6). **(C)** LDH assay. (*n* = 3) ***P < 0.01, vs. control group; ^#^*P* < 0.05, ^###^*P* < 0.01 vs. 10% normal serum group; ^□□□^*P* < 0.01 vs. 20% normal serum group; ^Δ^*P* < 0.05, ^ΔΔΔ^*P* < 0.01 vs. 30% normal serum group.

Effects of different concentrations of corticosterone (0, 5, 10, 50, 100, 200, 300, 400, 600, and 800 μM) were determined at different intervals (6, 12, 24, and 48 h) using CCK-8 assay to determine the optimal concentration and treatment time of corticosterone *in vitro* injury model. Treatment of primary hippocampal neurons with corticosterone at a low dose (0–50 μM) did not significantly decease cell viability compared with the control group (Figure 1A). Cell death was induced by high corticosterone doses (100–800 μM) in a concentration- and time-dependent manner. Reduction of cell viability to ~50% was achieved through treatment with 200 μM corticosterone for 24 h. Therefore, 200 μM corticosterone was chosen as the optimal concentration for subsequent experiments.

CCK-8 assay and LDH assay were used to measure cell viability and LDH release to determine the effect of SNS on corticosterone-induced damage in neurons. Primary hippocampal neurons were incubated with 10, 20, and 30% SNS-containing serum and 200 μM corticosterone for 24 h. Administration with 10, 20, and 30% SNS-containing serum significantly reversed corticosterone-induced decrease incell viability and increase in LDH release (Figures 1B,C). Notably, 20% SNS-containing serum had the highest neuroprotective effect, therefore it was used in subsequent experiments.

### Corticosterone Increases Autophagy and Inactivates the PI3K/Akt/mTOR Pathway in Primary Hippocampal Neurons

**Figure 2:**
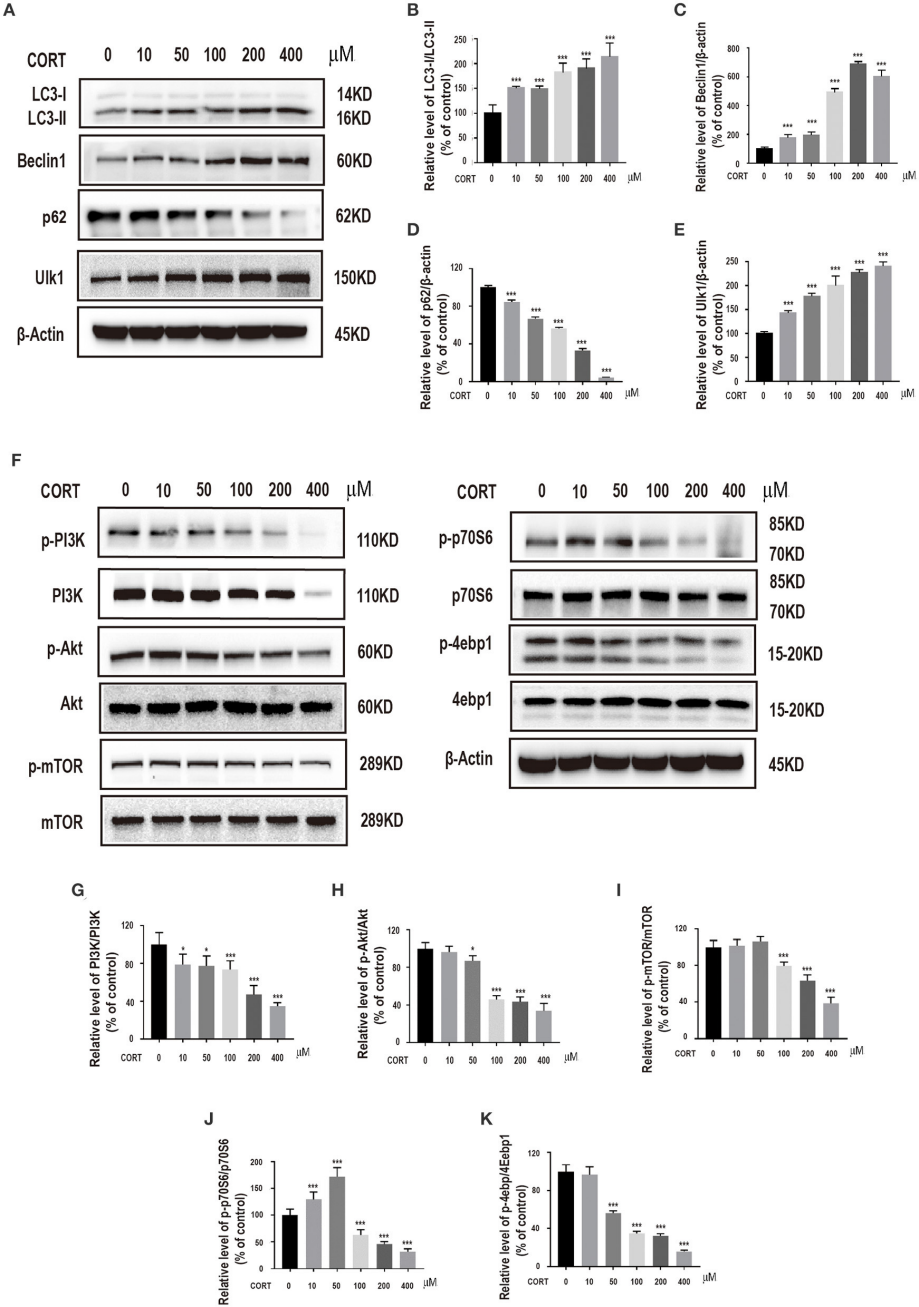
Incubation of cells with different concentrations of corticosterone (10, 50, 100, 200, 400 μM) for 24 h. **(A–E)** Western blot and quantitative analyses of LC3-II/LC3-I protein ratio, and Beclin1, p62 and ULK1 protein expression levels. **(F–K)** Western blot and quantitative analyses of PI3K, p-PI3K, Akt, p-Akt, mTOR, p-mTOR, p70S6, p-p70S6, 4ebp1 and p-4ebp1 protein expression. Each column represents mean ± SD (*n* = 3). *P < 0.05, ***P < 0.01 vs. control group.

To explore the role of autophagy on corticosterone-induced damage in primary hippocampal neurons, expression levels of specific intracellular autophagy-related proteins such as LC3-II/LC3-I, Beclin1, p62, ULK1, were determined. Prior to protein level analysis, different corticosterone concentrations (10, 50, 100, 200, and 400 μM) were administered for 24 h. Corticosterone administration significantly increased LC3-II/LC3-I ratio Beclin1 and ULK1 protein expression levels, and decreased p62 protein expression level in a concentration-dependent manner (Figures 2A–E). To explore the possible molecular mechanism of corticosterone in autophagy, PI3K, p-PI3K, Akt, p-Akt, mTOR, p-mTOR, p70S6, p-p70S6, 4ebp1 and p-4ebp1 protein expression levels were determined. Notably, corticosterone decreased p- PI3K/PI3K, p-Akt/Akt, p-mTOR/mTOR, p-p70S6/p70S6, and p-4ebp1/4ebp1 protein expression levels in a concentration- dependent manner (Figures 2F–K).

### SNS-Containing Serum Inhibits Autophagy in Corticosterone-Treated Primary Hippocampal Neurons

**Figure 3:**
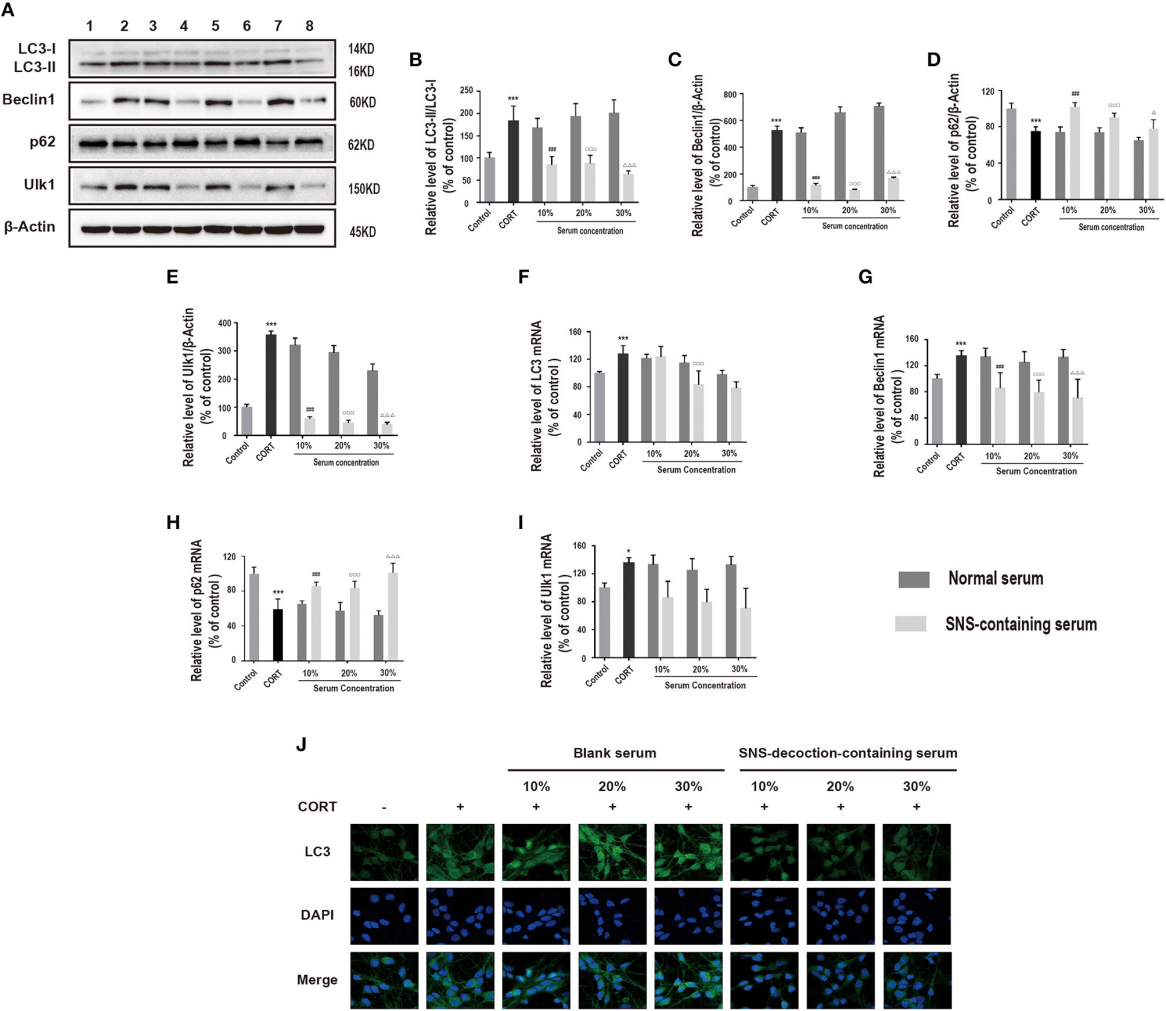
Cells were incubated with 200 μM corticosterone and SNS-containing serum for 24 h. **(A–E)** Western blot and quantitative analyses of LC3-II/LC3-I protein ratio, and Beclin1, p62 and ULK1 protein expression levels. (*n* = 3). **(F–I)** qRT-PCR analysis of LC3, Beclin1, p62 and ULK1 expression. (*n* = 3). **(J)** Immunofluorescence analysis of LC3 expression. *P < 0.05, ***P < 0.01 vs. control group; ^###^*P* < 0.01 vs. 10% normal serum group; ^▵^*P* < 0.05, ^▵▵▵^*P* < 0.01 vs. 20% normal serum group; ^ΔΔΔ^*P* < 0.01 vs. 30% normal serum group, (*n* = 3). 1:control group; 2: corticosterone group; 3: 10% normal serum + corticosterone group; 4: 10% SNS-containing serum + corticosterone group; 5: 20% normal serum + corticosterone group; 6: 20% SNS-containing serum + corticosterone group; 7: 30% normal serum + corticosterone group; 8:30% SNS-containing serum + corticosterone group.

To explore the effect of SNS on autophagy in corticosterone-induced injury, LC3-II/LC3-I protein ratio, Beclin1, p62 and ULK1 protein expression levels in hippocampal neurons treated with different concentrations of SNS-containing serum were determined. Administration of 10, 20, and 30% SNS-containing serum reduced LC3-II/LC3-I protein ratio, decreased Beclin1 and ULK1 protein expression levels, and increased protein expression level of p62 (Figures 3A–E). SNS-containing serum significantly decreased mRNA levels of LC3 and Beclin1, increased mRNA level of p62 (Figures 3F–H). In addition, SNS-containing serum down-regulated LC3 protein expression level (Figure 3I).

**Figure 4:**
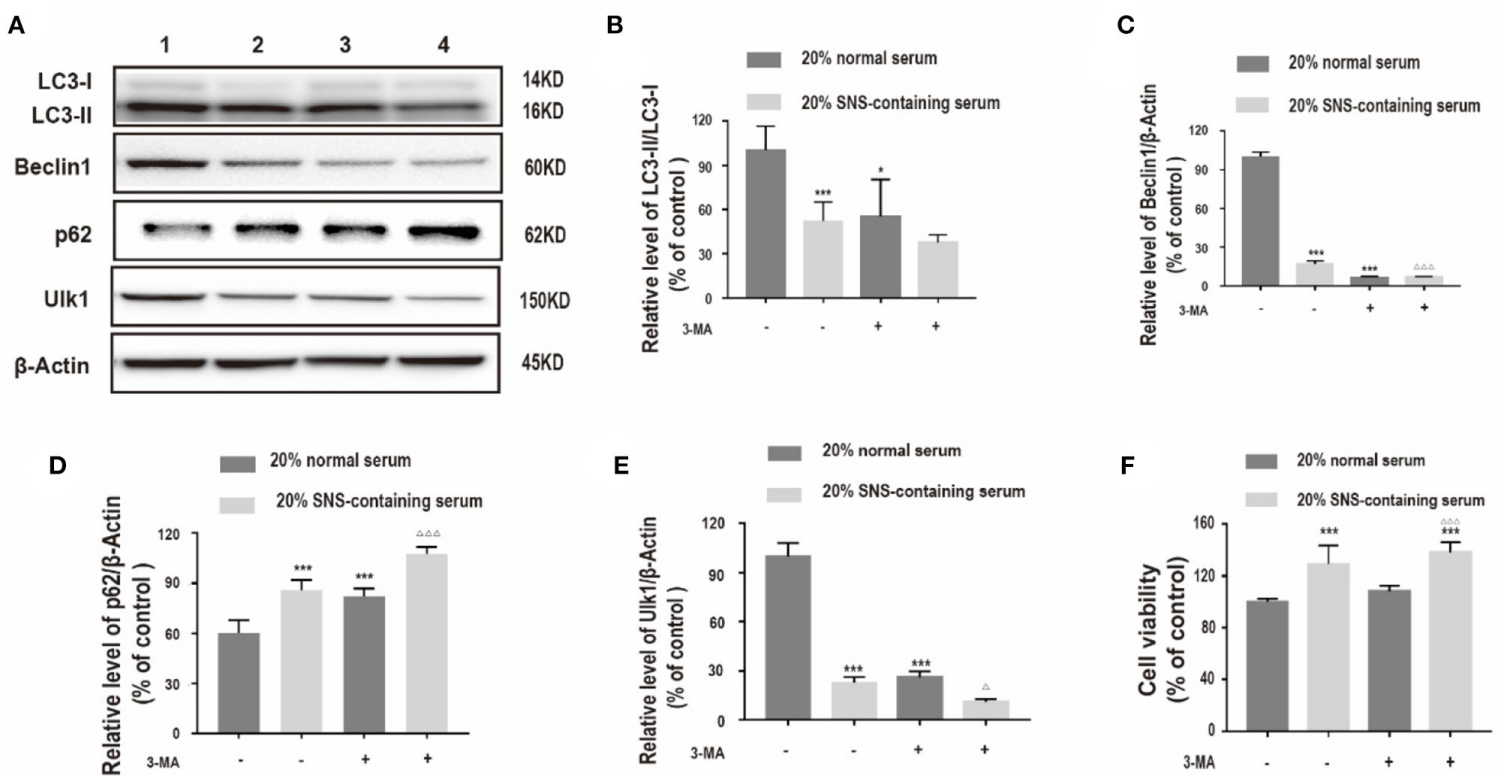
**(A–E)** Western blot and quantitative analyses of LC3II/LC3I ratio, and Beclin1, p62 and ULK1 protein expression levels. 1: 20% normal serum + corticosterone group; 2: 20% SNS-containing serum + corticosterone group; 3: 20% normal serum + corticosterone + 3-MA group; 4: 20% SNS-containing serum + 3-MA + corticosterone group. (*n* = 3). Each column represents mean ± SD. *P < 0.05, ***P < 0.01 vs. 20% normal serum + corticosterone group; ^Δ^*P* < 0.05, ^ΔΔΔ^*P* < 0.01 vs. 20% SNS-containing serum group. **(F)** cells were incubated with 200 μM corticosterone, 1mM 3-MA, and 20% serum for 24 h. CCK-8 assay was used to determine cell viability. ***P < 0.01 vs. 20% normal serum group, ^ΔΔΔ^*P* < 0.01 vs. 20% normal serum + 3-MA group (*n* = 4).

Further, the relationship between protective effect of SNS on corticosterone-treated neurons and downregulation of autophagy-related proteins was determined. Corticosterone-induced neurons exposed to SNS-containing serum were treated with 3-methyladenine (3-MA), an autophagy inhibitor. Analysis showed significant decrease in LC3-II/LC3-I protein ratio, and decrease in Beclin1, and ULK1 protein expression levels, increase in p62 protein expression level in the 3-MA-treated group compared with the levels in the group treated with 20% normal serum (Figures 4A–E). Furthermore, administration of 3-MA had no significant effect on neuronal viability, whereas a combination of 3-MA and 20% SNS-containing serum increased neuronal viability (Figure 4F).

### SNS-Containing Serum Activates PI3K/Akt/mTOR Pathway in Corticosterone-Induced Injury of Primary Hippocampal Neurons

**Figure 5:**
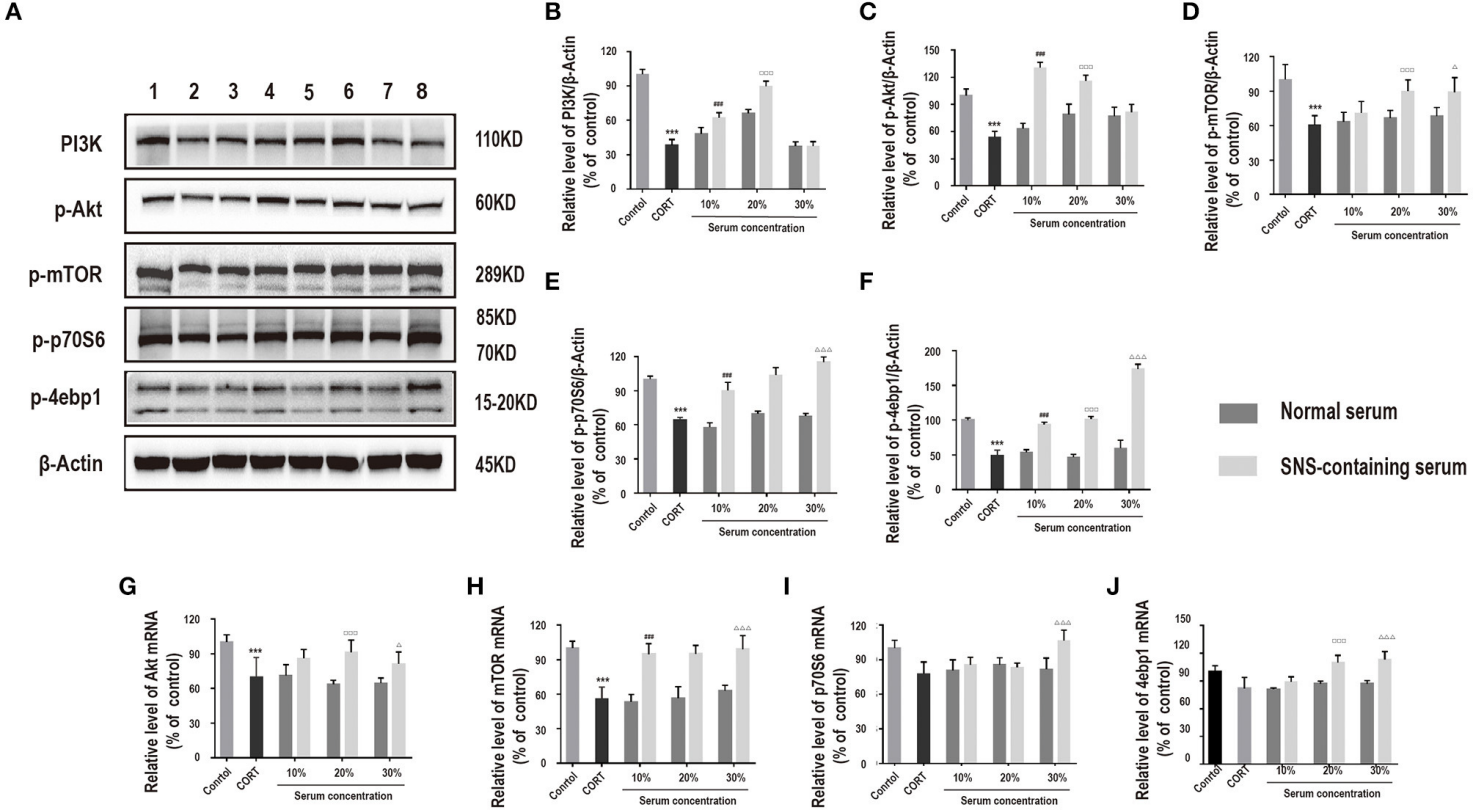
Cells were incubated with 200 μM corticosterone and SNS-containing serum for 24 h. **(A–F)** Western blot and quantitative analyses of PI3K, p-Akt, p-mTOR, p-p70S6, and p-4ebp1 protein, (*n* = 3). **(G–J)** qRT-PCR analysis of Akt, mTOR, p70S6, and 4ebp1 expression levels. ***P < 0.01 vs. control group; ^###^*P* < 0.01 vs. 10% normal serum group; ^□□□^*P* < 0.01 vs. 20% normal serum group; ^Δ^*P* < 0.05, ^ΔΔΔ^*P* < 0.01 vs. 30% normal serum group (*n* = 3).

PI3K/Akt/mTOR pathway plays an essential role in neuroprotection (17). Therefore, we hypothesized that activation of the PI3K/Akt/mTOR pathway may be involved in exerting neuroprotective effects of SNS against corticosterone. Protein expression levels of PI3K, p-Akt, p-mTOR, p-p70S6, and p-4ebp1 in corticosterone-induced primary hippocampal neurons treated with SNS-containing serum were determined. Analysis showed that SNS-containing serum significantly increased PI3K, p-AKT, p-mTOR, p-p70S6, and p-4ebp1 protein and mRNA expression levels (Figures 5A–J).

**Figure 6:**
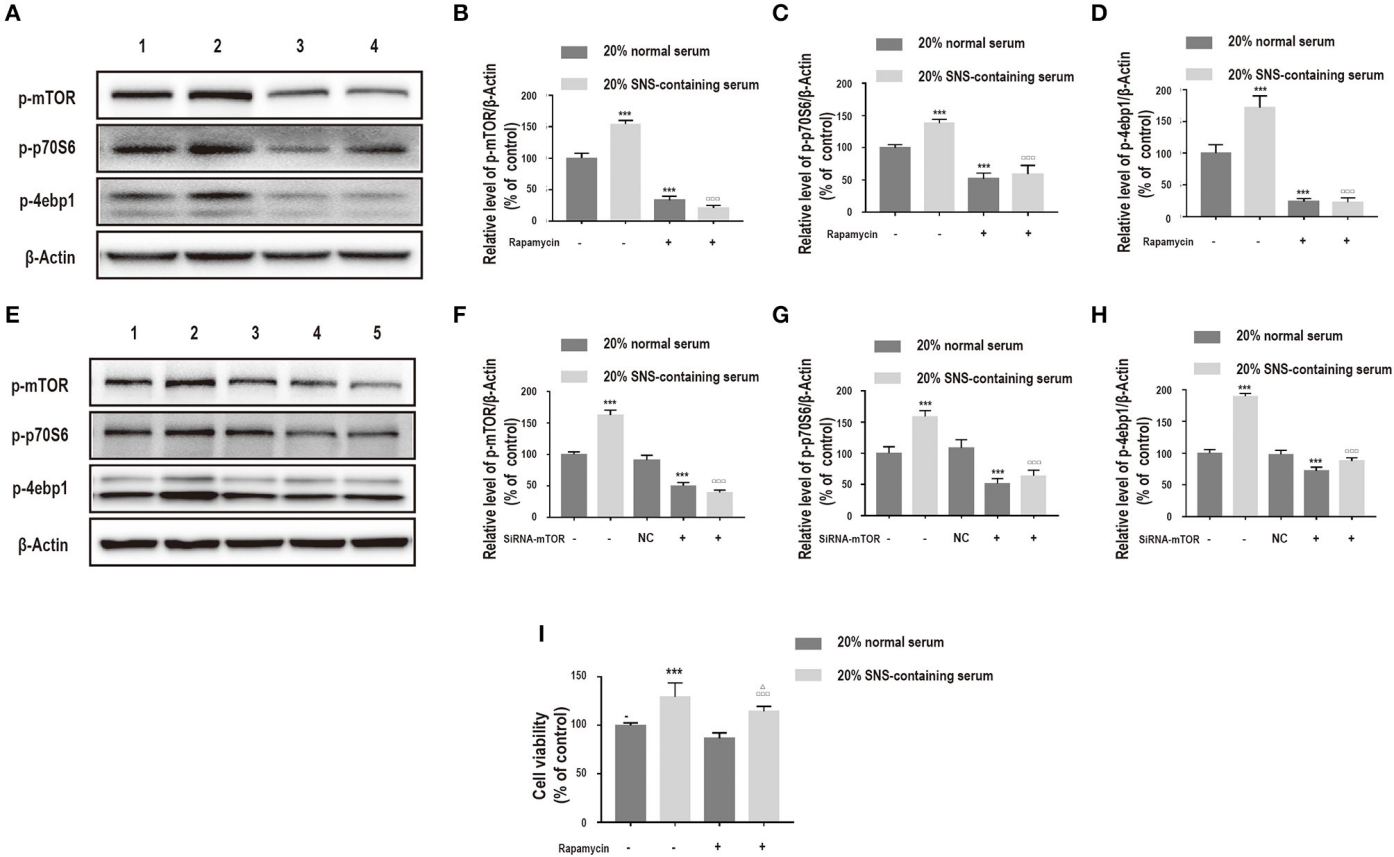
**(A–D)** Cells were incubated with 200 μM corticosterone, 1 μM rapamycin, and 20% serum for 24 h. Western blot and quantitative analyses of p-mTOR, p-p70S6, and p-4ebp1 expression. 1: 20% normal serum + corticosterone group; 2: 20% SNS-containing serum + corticosterone group; 3: 20% normal serum + corticosterone + rapamycin group; 4: 20% SNS-containing + corticosterone + rapamycin group. ***P < 0.01 vs. 20% normal serum + corticosterone group; ^□□□^*P* < 0.01 vs. 20% SNS-containing serum group (*n* = 3). **(E–H)** siRNA-mTOR transfected cells were incubated with 200 μM corticosterone and 20% SNS-containing serum for 24 h. Western blot and quantitative analyses of p-mTOR, p-p70S6, and p-4ebp1 protein expression levels. 1: 20% normal serum + corticosterone group; 2: 20% SNS-containing serum + corticosterone group; 3: 20% normal serum + corticosterone + siRNA-NC group; 4: 20% normal serum + corticosterone + siRNA-mTOR group; 5: 20% + SNS-containing serum + siRNA-mTOR group (*n* = 3). **(I)** CCK-8 assay for determination of cell viability. ***P < 0.01 vs. 20% normal serum group + corticosterone group; ^□□□^*P* < 0.01 vs. 20% normal serum+ corticosterone +rapamycin;^Δ^*P* < 0.05 vs. 20% SNS-containing serum group+ corticosterone group (*n* = 4).

Notably, the mTOR inhibitor, rapamycin and siRNA-mTOR significantly decreased p-mTOR, p-p70S6, and p-4ebp1 protein levels (Figures 6A–H), implying that the PI3K/Akt/mTOR pathway was induced by SNS-containing serum. Further, to explore the role of PI3K/Akt/mTOR pathway on SNS-mediated neuroprotection of corticosterone-induced neurons, cells were treated with rapamycin. Administration of 20% SNS-containing serum increased neuronal cell viability, whereas neuronal viability decreased in rapamycin +20% SNS-containing serum group compared with 20% SNS-containing serum group (Figure 6I), (–).

## Discussion

In this study, pharmacological and genetic tools were used to explore the mechanisms of corticosterone-induced injury and the protective effects of SNS in primary hippocampal neurons. The findings of this study show that SNS protects neurons against corticosterone-induced injury by inhibiting autophagy through induction of PI3K/AKT/mTOR pathway. These findings provides a basis for development of a therapeutic agent for prevention or treatment of corticosterone-induced neuronal injury.

Exogenous administration of high doses of corticosterone causes depression and neuronal damage in rodents, thus affecting cognitive functions. Corticosterone is used to establish models of *in vitro* and *in vivo* depression (18, 19). In this study, 100–800 μM corticosterone caused neuronal damage *in vitro*, which is consistent with findings from previous studies that exposure to high doses of corticosterone is required to cause significant neurotoxicity in primary hippocampal neurons (20). On the contrary, Liu et al. reported that 1 μM corticosterone is sufficient to cause effects in neuronal survival (21). This difference in susceptibility might be because, unlike hippocampal neurons, astrocytes are more prone to injury resulting from corticosterone-induced apoptosis, have lower levels of reactive oxygen species and are more resistant to cytotoxic effects of corticosterone (22).

TCM is used for treatment of depression in China for centuries and is currently widely used in Western countries. In addition, drug-containing serum allows study of therapeutic effects of TCM. Normal serum subgroup and SNS-containing serum subgroup were included for comparison to rule out the effect of serum on experimental results (23). SNS is a traditional Chinese herbal formula used for treatment of depression. Li Y et al. reported that SNS is more effective in reducing depression-associated symptoms compared with use of fluoxetine (24). The main active chemical components in SNS include paeoniflorin and saikosaponin. Pretreatment with paeoniflorin prevents cell death in glutamate-induced PC12 cells (25). In addition, saikosaponin alleviates corticosterone-induced neurotoxicity in PC12 cells in a dose dependent manner. Notably, 10, 20, and 30% SNS-containing serum increased survival rate and inhibited LDH release in corticosterone-induced neurons. This finding shows that SNS protects primary hippocampal neurons against neurotoxicity induced by corticosterone. Therefore, SNS exerts antidepressant-like effects *in vitro*.

Previous studies reported that depression is associated with autophagy. Several autophagy related genes (Atgs) play key roles in the process of autophagy. Beclin1 is implicated in autophagosome formation, and studies reported that the level of autophagy is up-regulated by over-expression of Beclin1. LC3-I is lipidated to form LC3-II on autophagosomal membranes. LC3-II plays a key role in formation of autophagosomal membranes, therefore, LC3-II /LC3-I is an effective indicator of the level of autophagy activity. p62 is an autophagic adapter that mediates selective recognition and degradation of specific autophagy substrates, and is widely used as a marker to monitor autophagic flux (26–28). In addition, ULK1-FIP200-Atg13 complex formation is a characteristic of the initial stage of autophagy. Previous studies reported that paeoniflorin effectively protects PC12 cells from autophagic pathways (29), and saikosaponin D and A prevent autophagy after EV-A71 infection (30). These findings indicate that the protective function of SNS in depression may be correlated with autophagy.

In this study, administration of corticosterone reduced LC3-II/LC3-I protein ratio, decreased the levels of Beclin1 and ULK1 protein, increased p62 protein expression level in a dose-dependent manner. This finding implies that corticosterone treatment induces autophagy in primary hippocampal neurons. Similar findings were reported previously that corticosterone exposure causes a dose-dependent increase in LC3-II expression in PC12 cells (31). However, a recent study reported a dose-dependent decrease in autophagy in prefrontal cortex and hippocampus of CUMS mice (32). The differences in results can be attributed to differences in cell types and stimuli used. SNS-containing serum, 3-MA, and a combination of SNS-containing serum and 3-MA significantly decreased LC3-II/LC3-I protein ratio, decreased Beclin1, and ULK1 and increased p62 protein expression levels. Notably, treatment with SNS serum-3-MA combination showed the highest effects. These findings indicate that SNS inhibits excessive autophagy in corticosterone-injured neurons.

PI3K/AKT/mTOR pathway regulates protein synthesis, cell cycle, and cell metabolism by phosphorylating downstream proteins thus modulating cell growth, proliferation, apoptosis, and autophagy (33). mRNA expression of AKT1 and mTOR are downregulated in bipolar depression, and may induce autophagy (34). Reduced mTOR signaling is reported in major depressive disorder, compared with healthy controls (35). mTOR pathway is a major modulator of autophagy. PI3K and AKT are upstream targets of mTOR, which are activated by receptors of neurotrophins and growth factors. Activation of mTOR induces activation of two major downstream substrates, p70S6 and p-4ebp1, resulting in induction of protein translation (36, 37). Cui L et al. reported that saikosaponin thus inhibits autophagy in pancreatic fibrosis through regulation of PI3K/Akt/mTOR pathway (38). Therefore, we hypothesized that PI3K/Akt/mTOR pathway is a major target of SNS in prevention of excessive autophagy in corticosterone-induced neurons.

In this study, 100–400 μM corticosterone decreased expression of PI3K, p-Akt, p-mTOR, p-p70S6, and p-4ebp1, indicating that PI3K/Akt/mTOR pathway is activated by treatment with corticosterone. The findings of this study show that corticosterone activates neuronal autophagy and inhibits PI3K/AKT/mTOR pathway. Treatment with SNS-containing serum increased PI3K, p-AKT, p-mTOR, p-p70S6, and p-4ebp1 protein and mRNA expression levels in corticosterone-induced neurons. Notably, rapamycin and siRNA-mTOR significantly reduced protein levels of these autophagy-related factors. This finding implies that SNS activates the PI3K/Akt/mTOR pathway. Further, Administration of 20% SNS-containing serum increased neuronal cell viability, whereas neuronal viability decreased in rapamycin +20% SNS-containing serum group compared with 20% SNS-containing serum group,indicating that SNS protects neurons against damage through modulation of the PI3K/Akt/mTOR pathway. 3-MA is a selective inhibitor of PI3K, however, it had no significant effects on increase in neuronal viability, whereas a combination of 3-MA and 20% SNS-containing serum increased neuronal viability. This observation may be because other pathways, such as AMPK/mTOR signaling pathway in addition to PI3K/Akt/mTOR pathway may be involved in autophagy activated by corticosterone (39) which are then modulated by SNS.

Autophagy is a complex process and SNS comprises various active components, therefore, further studies should explore the effects and mechanisms of SNS-containing serum. In addition, qualitative and quantitative profiling of SNS should be performed using high performance liquid chromatography (HPLC). Furthermore, a limitation of this study is that anti-depression mechanism of SNS was only explored through *in vitro* experiment. However, a previous study by our group using CUMS model reported that SNS activates PI3K/ Akt /mTOR pathway and reduces autophagy in hippocampus of rats which is consistent with the findings of the current study (40).

## Conclusions

In summary, the findings of this study show that 200 μM corticosterone decreases survival rate, activates autophagy, and inhibits PI3K/Akt/mTOR pathway in corticosterone-induced neuronal injury. SNS-containing serum inhibits autophagy through the PI3K/Akt/mTOR pathway. These findings provide information on the mechanism of SNS activity on depression, and provide a theoretical basis for future application of SNS in clinical treatment of depression. Further studies should be conducted to optimize the clinical use of SNS in management of neurodegenerative complications.

## Data Availability Statement

The original contributions presented in the study are included in the article/supplementary material, further inquiries can be directed to the corresponding authors.

## Ethics Statement

The animal study was reviewed and approved by the Animal Care and Committee of Guangzhou University of Chinese Medicine.

## Author Contributions

MZ performed the experiments and wrote the manuscript. HA and SH conceptualized and designed the experiments. YZ organized generation, collection, assembly, and interpretation of data. HN supplemented experimental data. HS, JS, XY, WC, WZ, XZ, and CH performed the experiments. All authors contributed to the article and approved the submitted version.

## Conflict of Interest

The authors declare that the research was conducted in the absence of any commercial or financial relationships that could be construed as a potential conflict of interest.
